# Listening to Music and Playing Activities during Recreation between Lessons Regenerate Children’s Cognitive Performance at Different Times of Day

**DOI:** 10.3390/children9101587

**Published:** 2022-10-20

**Authors:** Nourhen Mezghani, Achraf Ammar, Turki Mohsen Alzahrani, Atyh Hadadi, Salma Abedelmalek, Omar Trabelsi, Sabeh ben Abdallah, Cyrine H’mida, Omar Boukhris, Liwa Masmoudi, Khaled Trabelsi, Hamdi Chtourou

**Affiliations:** 1 Department of Sport Sciences, College of Education, Taif University, P.O. Box 11099, Taif 21944, Saudi Arabia; 2 Department of Training and Movement Science, Institute of Sport Science, Johannes Gutenberg-University Mainz, 55122 Mainz, Germany; 3 Interdisciplinary Laboratory in Neurosciences, Physiology and Psychology: Physical Activity, Health and Learning (LINP2), UFR STAPS (Faculty of Sport Sciences), UPL, Paris Nanterre University, 92000 Nanterre, France; 4 Higher Institute of Sport and Physical Education of Sfax, University of Sfax, Sfax 3000, Tunisia; 5 Department of Physiology and Functional Explorations, Sousse Faculty of Medicine, Sousse 4000, Tunisia; 6 Department of Sport Sciences and Physical Education, University of Education, Hail 55436, Saudi Arabia; 7 Research Laboratory, Education, Motricity, Sport and Health (EM2S), LR15JS01, High Institute of Sport and Physical Education, University of Sfax, Sfax 3000, Tunisia; 8 Research Unit: Physical Activity, Sport, and Health, UR18JS01, National Observatory of Sport, Tunis 1003, Tunisia; 9 Sport and Exercise Science, School of Allied Health, Human Services and Sport, La Trobe University, Melbourne 3086, Australia

**Keywords:** diurnal variation, cognitive performance, physical activity, children

## Abstract

The interruption of learning processes by breaks filled with diverse activities is common in everyday life. The purpose of this study was to investigate the effects of the time of day (TOD) of playing with or without music during recess on cognitive performance regeneration among students aged between 11 and 12. Twenty-seven volunteer children (aged 12 ± 0.6 years) completed four trials at 09h45, 10h15, 14h45, and 15h15. Each test session was separated by recreation between classroom lessons with different conditions: music only (MSC), playing only (PAY), music and playing (MSC-PAY), and without music and playing (NON). During each session, oral temperature, reaction time (RT), and constant attention (CA) were measured. For all parameters, the ANOVA revealed a significant effect of the type of activity. However, no significant effect of the TOD and no significant interaction of type of activity × TOD were reported. The Bonferroni post hoc test revealed that Δ-core temperature was significantly higher during PAY and MSC-PAY compared to NON (*p* < 0.05). Δ-attention was higher during MSC, PAY, and MSC-PAY compared to NON (*p* < 0.001). Δ-attention was lower during MSC (*p* < 0.05), PAY (*p* < 0.05), and MSC-PAY (*p* < 0.01) compared to NON. Therefore, playing, listening to music, and playing while listening to music at recess improve the child’s ability to regenerate cognitive performance regardless of the TOD.

## 1. Introduction

One of the most pressing challenges for any society in the 21st century is to find effective ways to improve cognitive and academic performance, increase physical activity levels, and reduce sedentary behaviors in children. Children are spending more time indoors while pediatric, mental, and behavioral health problems are increasing.

School rhythms can be influenced by many factors related to the type of tasks, their intensity, and the conditions of their execution [1]. Several studies have reported that children’s physical and cognitive performance is influenced by the time of day (TOD) [2,3,4]. Masmoudi et al. [2] and Elghoul et al. [3] reported the existence of a diurnal variation in children’s dribbling and dart-throwing performances with higher values in the afternoon. Similarly, Jarraya et al. [4] showed that cognitive performance (i.e., reaction time (RT) and constant attention (CA)) depends on the TOD, with higher values around the daily peak of core temperature. However, before and after an anatomy lesson with university students, H’mida et al. [5] reported that attention was better in the morning. Regardless of the geographical location of the school [6], the level of cognitive performance at school is marked by low values at the beginning of the school day, an improvement until mid- or late-morning, a relapse after the lunch break (post-prandial decline), and then another increase in the academic performance in the afternoon [6,7,8]. Consequently, Testu [8] indicates that school time must take into account the child’s chrono-psychological rhythms (i.e., to allow for better opportunity for learning). Furthermore, the circadian rhythm of core temperature is frequently used as an indicator of the biological clock as a result of its strong endogenous determination [9]. Indeed, cognitive performances, including simple RTs and mental states, have been shown to be rhythmic with the core temperature’s circadian cycle. However, the rhythms of mood states and complex mental tasks may not coincide with those of core temperature [9].

The middle childhood period extends from the age of 6 years to 12 years and continues until the beginning of puberty. At this age, children are more oriented towards more organized or ritualized games and sports. The work of middle childhood is to develop academic success, intellectual skills, and physical abilities. Play is a break from the demands of the real world. Recess is the best example of the transition from reality to imaginary play. In addition, parents often give their children time to play and to unload the demands of the day before starting their homework [10].

In the classroom, lengthening the time spent in lessons does not automatically translate into more time spent on learning [11]. Thus, recess is important for effective learning during the child’s school day. They allow them to rest, play, imagine, think, move, and specialize [11]. Taking a break from learning can help consolidate memories and prepare for upcoming tasks [12]. Breaks are often occupied by different activities, such as playing games or listening to music, and the effects of break activities on learning remain to be explored [13]. Mobile games have been shown to develop children’s creativity and socialization, as they can be the starting point for many learning situations [14]. Play provides opportunities for the development of cognitive skills throughout a child’s development. Play allows for reasoning and expressing emotions, conceiving other ways of looking at a situation, looking at new ways of reacting to situations, letting the imagination run wild, and assimilating cause-and-effect relationships [10].

Recently, Mezghanni et al. [15] investigated the relationship between cognitive performance, recess play, and time of day. The authors revealed that recess improves children’s ability to regenerate cognitive performance independent of the time of day. In addition to physical exercise, Su et al. [16] showed that learning with background music generates increased attention. The authors postulated that listening to background music seems to have a positive effect that could influence cognitive performance and improve learning. Probably the best-known approach in this field is the so-called Mozart effect [17]. Rauscher et al. [17] found that listening to a Mozart sonata increases spatial reasoning ability. In addition, learning with background music has recently gained more interest due to advances in music technology [18]. Music therapy is a modern technique that emerged in the mid-twentieth century, primarily designed to promote emotional well-being and facilitate social group integration [19,20] by stimulating complex emotional and sensorimotor receptors [21,22]. Similarly, music has been widely used to promote learning and physical performance [23,24,25]. In this context, Thompson et al. [26] revealed that the tempo and intensity of music influence learning outcomes, with positive effect being associated with fast-and-soft music and impeded learning with slow-and-loud music or fast-and-loud music. In addition, Yerkes-Dodson [27] proved the law of the inverted U-shape, establishing optimal arousal in a learning situation. Thus, learners with a low level of arousal are not engaged enough to invest in the learning process, while too high a level of arousal can cause distracting feelings such as anxiety. Student engagement has long been recognized in educational research as a primary facilitator of academic success [28]. Research findings on the relationship between background music and academic success are controversial. While some have found no effect of background music [29,30], others have proven that background music has a negative [31,32] or positive [33,34] impact on learning outcomes. Hall [35] indicated that combining music and motor activities decreases the students’ transition times and increases their engagement, providing a valuable enhancement to instructional time.

To date, to the best of the authors’ knowledge, the relationship between music and motor activities during recreation and the diurnal rhythm of cognitive performance has not yet been examined in children. However, previous studies reported that the effect of music on physical performance is time-of-day dependent [36,37]. Therefore, the present study examined the effect of three different activities during recreation (i.e., listening to music only (MSC), playing only (PAY), listening to music and playing (MSC-PAY), and without any music and playing (NON)) on the intra-day variation of cognitive performances in children aged 11–12 years old.

## 2. Materials and Methods

### 2.1. Participants

Twenty-seven children with an average age of 12 ± 0.6 years, an average body height of 146 ± 5.2 cm, and an average body mass of 37.2 ± 2.9 kg participated voluntarily in this study. Before data collection, permission to conduct the study was obtained from the school’s principal and head teachers and consent forms were obtained from participants’ parents or guardians. All participants were chosen from a medium socio-economic level, and were near pubescent, based on standard criteria [38]. 

The project was approved by the University’s Institutional Review Board before the conduction of the study according to the Declaration of Helsinki. The participants were also selected based on their answers to the self-assessment questionnaire “Children’s Morningness–Eveningness Preferences (CMEP, [39])”. Only “morning types were involved in the present study to have a sample without extreme late chronotype”. Based on the KIDMED, all subjects had the same quality of diet [40]. Based on the Pittsburgh Sleep Quality Index, all subjects had regular sleep schedules, with an average wake-up time of 06h00 ± 00h41 and they went to bed at 21h00 ± 00h22. 

### 2.2. Experimental Design

After an initial familiarization session, where all test procedures were explained and practiced, subjects were randomized into four groups. Four test sessions were randomly performed at the following times of day: 09h45, 10h15, 14h45, and 15h15, during four successive Thursdays. For each session, the participant engaged in one single type of activity during recreation: MSC, PAY, MSC-PAY, or NON. The four groups of students alternated the four types of activities during the four consecutive Thursdays.

Thursday was chosen because it is the day on which school performance is better [41]. Participants performed the reaction time (RT) test followed by the digit cancellation test during each test session. A clinical digital thermometer (Omron^®^, Paris, France; accuracy ±0.05 °C) was inserted sublingually for 3 min at the beginning of each test session to measure the oral temperature. Subjects were asked to maintain their usual sleeping habits on the night preceding each test session, with a minimum of 8 sleeping hours. Moreover, on the day before each test session, they were requested to avoid strenuous activity and maintain their habitual physical activity. Compliance with these directions was verified in an interview before each session. During the entire experimental period, the average relative humidity and ambient temperature of the classroom (44.3 ± 7.6% and 21.1 ± 1.1 °C, respectively) were stable.

### 2.3. Procedures and Break Activity Scenarios

Participants were instructed to “stay idle, play, listen to music, or play and listen to music”. Immediately after the break, participants’ performance was assessed. In the present study, the 10 min break duration was based on the length of the music and was in line with the previous break range of 5 to 20 min [12,42,43]. In previous investigations, Mozart’s Sonata KV.448 was used as major musical piece to assess the effects of music on cognitive function [44,45].

### 2.4. Break Activity Scenarios

To evaluate the effects of different break activities on the inability to refocus cognitively, break activity scenarios were used. Participants were instructed to engage in two types of play consecutively (the game of hunter and fishing game), listening to music (through headphones: Mozart’s Sonata for Two Pianos in D Major, KV.448—Allegro con spirit), playing and listening to music together, or they were instructed to rest without activity [44].

### 2.5. Games Practiced during Recess

We delimited a large playground, we arranged the school class, and the pupils participated in two types of play consecutively. The games were selected according to the pupil’s preference to promote fun during recreation. These games are very enthusiastic and encourage students to complete, as fast as possible, large, whole-body movements.

### 2.6. The Game of Hunter

A hunter is nominated from the participating pupils and must catch one of his colleagues in a limited space of 100 m^2^. Both hunters run (hand in hand) together to catch the 3rd hunter and so on until, finally, a large belt is formed. The winner is the last remaining student. 

### 2.7. The Fishing Game 

Pupils raise their arms to make a round after they have secretly decided when to lower them (i.e., when saying a specific word in a song or at a specific number during an enumeration). At the set time, the other pupils move in and out of the round; the others are caught inside when the kids’ arms are lowered as they sing “Join the round, we’ve got you!”

### 2.8. Mozart’s Sonata for Two Pianos in D Major, KV.448

The Sonata for Two Pianos in D major, KV. 448, was composed in the Galant style in 1781 by Wolfgang Amadeus Mozart. It was written with three movements in sonata-allegro form. This sonata with interlocking melodies and simultaneous cadences has been also used to test the theory of the Mozart effect, suggesting that, compared to other kinds of music, classical increases brain activity [43,46].

### 2.9. The Digit Cancellation Test

The digit cancellation test (i.e., a paper-pencil test) is a psychometric task that measures constant attention [47]. As previously described by Jarraya et al. [4], the participant has to scan a sequence of numbers and strike out the “target number” that varies with each form of the test (5 different forms have been used) and is shown at the top of the page. The test lasts 1 min and the score is calculated as number of correct responses. Higher scores reflect better performance.

### 2.10. Reaction Time (RT)

The reaction time test, notably the simple reaction time assessed via the NPRI software (version 4.05) [25], was used to reflect individual motor performance [8]. The test consists of responding to a visual stimulus (the appearance of the word “NOW” on the computer screen) by clicking on a computer key as quickly as possible. The software then calculates the time between the stimulus appearance and the response (in ms). An average of 10 trials was recorded for analysis and used for the calculation.

### 2.11. Statistical Analysis

All statistical tests were processed using Statistica Software (StatSoft 7.1, Paris, France). Data are reported as mean ± SD (standard deviation). The assumption of normality was confirmed using the Kolmogorov–Smirnov test of normality A comparison of mean data was analyzed using a two-way analysis of variance ANOVA. When a significant difference was determined, pairwise comparisons were performed using the Bonferroni test. Statistical significance was set at *p* < 0.05.

## 3. Results

**Figure 1:**
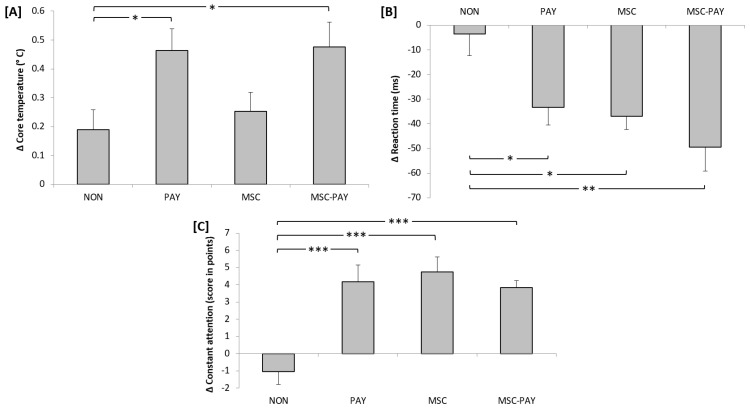
Delta changes in core temperature (**A**), reaction time (**B**), and constant attention (**C**) after four types of activity at recess. * for *p* < 0.05; ** for *p* < 0.01 and *** for *p* < 0.001.

Figure 1 shows the Δ changes in core temperature, AC, and RT recorded after the four types of recess (MSC, PAY, MSC-PAY, and NO), regardless of the time of day, and the comparison between the Δ changes after the three recesses with activity (MSC, PAY, MSC-PAY) and the one without (NON).

### 3.1. Oral Temperature

Statistical analysis revealed no significant effect of TOD (F(1, 26) = 0.15; *p* = 0.7; $\eta_{p}^{2}$ = 0.006) and no significant interaction of type of activity × TOD F(3, 78) = 0.02; *p* = 0.995; $\eta_{p}^{2}$ = 0.001. However, a significant effect of type of activity (F(3, 78) = 3.2; *p* = 0.028; $\eta_{p}^{2}$ = 0.109) was reported. The Bonferroni post hoc test showed that the Δ-core temperature was significantly higher during PAY and MSC-PAY compared to NON (*p* < 0.05).

### 3.2. Attention

The ANOVA revealed a significant effect of type of activity (F(3, 78) = 12.43; *p* < 0,001; $\eta_{p}^{2}$ = 0.323. However, no significant effect of TOD (F(1, 26) = 0.66; *p* = 0.426; $\eta_{p}^{2}$ = 0.025) and no significant interaction of type of activity × TOD (F(3, 78) = 0.61; *p* = 0.609; $\eta_{p}^{2}$ = 0.023) were registered. The post hoc tests showed that Δ-attention was higher during MSC, PAY, and MSC-PAY compared to NON (*p* < 0.001).

### 3.3. The Reaction Time (RT)

The ANOVA revealed a significant effect of type of activity (F(3, 78) = 5.54; *p* = 0.002; $\eta_{p}^{2}$ = 0.176). However, no significant effect of TOD (F(1, 26) = 1.18; *p* = 0.287; $\eta_{p}^{2}$ = 0.043) and no significant interaction of type of activity × TOD F(3, 78) = 1.16; *p* = 0.33; $\eta_{p}^{2}$ = 0.043) were noted. The post hoc tests showed that Δ-RT was lower during MSC (*p* < 0.05), PAY (*p* < 0.05), and MSC-PAY (*p* < 0.01) compared to NON, indicating that children were faster during these active conditions.

## 4. Discussions

The present study aimed to investigate the effect of different break activities (i.e., MSC, PAY, MSC-PAY, and NON) on cognitive performance (i.e., RT and CA) during recess realized at different TOD in pupils aged 11–12 years.

The main results showed no significant effect of TOD on children’s core temperature and cognitive performance (i.e., RT and CA). Contrarily, previous studies revealed that cognitive performances are time-of-day dependent, with either higher values at the end of the afternoon, around the daily peak of core temperature [3,4,8], or an earlier peak in the morning, with more marked fall afterward for more “complex” tasks involving larger cognitive components [48]. Recess is at the heart of today’s debate over the role of schools in recognizing alterations in children’s performance. The results of the present study showed that children’s cognitive performance can be restored following an active recess regardless of the TOD. These findings are in line with the suggestions of the American Academy of Pediatrics [49], indicating that recess is an essential and necessary part of a child’s development. 

Regarding the effect of the recess condition, the present findings indicate a significant effect on core temperature, with higher Δ-core temperatures during PAY and MSC-PAY compared to NO. This temperature elevation during a physically active recess is due to the energy released by the living organism following physical activity. The results also showed a significant effect of the recess condition on cognitive functions and demonstrated an improved RT and CA following an active recess (i.e., playing, hearing music, playing music) compared to a recess without activity. These findings are in agreement with the suggestions of the American Academy of Pediatrics [49], which state that any type of activity at recess benefits cognitive performance afterwards. However, Stellino and Sinclair [50] reported that the student’s ability to refocus on the cognitive level was more stimulated by the break in class than by the mode of activity that occurred during this recess. 

The present results support previous reports on the potential positive effects of background music on students’ intrinsic motivation and lesson satisfaction. In fact, music seems to have a positive and stimulating effect [51,52], which could improve learning. Previous studies revealed that background music has positive effects on arousal and mood, which, in turn, affect learning outcomes [53,54]. Additionally, the music’s mediation effect has been reported to influence spatial abilities and cognitive performance [53,54] due to exposure to a pleasant stimulus [51]. Consequently, appropriate background music in the right tempo and style could be beneficial by bringing the learner’s mood and arousal level to an optimal state, thereby promoting the learning process.

The results showed a significant effect of the activity with improved attention during an active recess compared to a non-active recess. In accordance with these findings, previous studies found that recesses of 20 or 60 min per day enhanced classroom attention [49]. The slight decline in attention reported in the present study after a non-active recess is probably due to an increased level of stress as a result of inactivity and restricted interacting and wandering, which a child naturally enjoys. 

In the same context, Stevenson and Stigler [55] showed that Japanese and Chinese students, known as one of best achievers worldwide, attend schools that offer short breaks every 50 min.

Therefore, it is worth noting that enough play-based recess periods, which allow children to regain their concentration before lessons resume, must be scheduled at regular intervals throughout the school day [56]. Additionally, a previous study showed that kids in schools pay more attention to teachers after they have had an unstructured break in which they are free to play [14].

In the same context, music is tightly connected to the attention system [57], and it can temporarily improve cognitive performance (e.g., those associated with attentional processes) and memory and learning via increased arousal [57]. Indeed, 1 hour of daily listening to one’s favorite music for 2 months was shown to significantly enhance the recovery of attentional processes in acute post-stroke participants [57]. Interestingly, in those with left hemisphere stroke, this improvement in attentional processes was also associated with a significant increase in prefrontal gray matter volume [58].

Furthermore, attention, which involves inhibition and impulse control, is highly valued by parents due to it enhancing learning. Previous studies showed that physically active children have better learning abilities and classroom behaviors compared to sedentary peers [56,59]. Therefore, the association between physical activity and music and cognitive performance for early elementary-age schoolchildren is not surprising. For example, the patty-cake game, which combines clapping the hands and singing an English nursery rhyme and involves synchrony across motor, proprioceptive, tactile, and auditory inputs, was shown to enhance classroom learning [56]. Similarly, problem-solving-based games have been suggested to promote higher skill levels, notably the executive function, which integrates attention with other cognitive functions (i.e., organizing, sequencing, planning, and decision making) [56].

Due to its complex process, including motor control, coordination of multiple sensory modalities, attentional shifting, monitoring, inhibition, and working memory, the fishing game performed by schoolchild can also have beneficial effects on the recovery of their cognitive performance [56]. Although it has been the subject of few scientific studies in young children, the association between play and music has the potential to be used in techniques for stimulating reaction time and constant attention, which are central to recovering the cognitive performance associated with academic achievement.

In addition, kids are more likely to perform physical activity through play, which is also essential for their physical, cognitive, emotional, and social development [60,61]. To help reduce the risk of obesity, the American Academy of Pediatrics policy recommended 60 min of moderate to vigorous activity per day [62]. Even minor movement during recess can counterbalance sedentary time at school and home and helps the schoolchild achieve the recommended daily PA.

**Strength and Limitation:** To the best of the authors’ knowledge, this is the first study to assess the combined effect of playing and listening to music on children’s cognitive performances and their diurnal variations. However, there were also limitations related to the play and music contents and the tested cognitive abilities. Therefore, the promising findings need to be interpreted with caution. Further large studies in different time zones and including more general play and music contents and a more comprehensive cognitive ability test battery are needed to confirm our preliminary findings.

## 5. Conclusions

The school represents a unique opportunity to enhance children’s physical fitness. Thus, minimizing or eliminating activities (e.g., playing and/or listening to music) during recess may be counterproductive for academic achievement. It needs to be emphasized that these recess activities are necessary for optimal brain development in schoolchildren. Recess promotes both physical health and social development, as well as children’s cognitive performance and academic success [60,63]. Therefore, schools should enthusiastically promote play for its traditional merit, as playing allows children to experience the joys of movement, friendship and creativity. In the present study, we showed that any type of challenging activity (play and/or listening to music) that improves emotional expression at recess has a potent benefit on the ability to regenerate cognitive performance (i.e., RT and CA) regardless of the TOD.

## 6. Practical Recommendations

To optimize a child’s physical and cognitive development, recess should be seen as a vital part of a child’s time, and it should not be withheld for academic or punitive reasons. Some kinds of childhood activities (playing, hearing music, playing and hearing music) should be considered as a form of pleasant stimulus and a typical behavior to help develop certain brain networks. Moreover, they should be seen as necessary to successfully build classroom skills while improving energy balance and preventing obesity.

## Data Availability

Data are available from the corresponding author upon reasonable request.
